# Full-Length Transcriptome of *Thalassiosira* *weissflogii* as a Reference Resource and Mining of Chitin-Related Genes

**DOI:** 10.3390/md19070392

**Published:** 2021-07-13

**Authors:** Haomiao Cheng, Chris Bowler, Xiaohui Xing, Vincent Bulone, Zhanru Shao, Delin Duan

**Affiliations:** 1 CAS and Shandong Province Key Laboratory of Experimental Marine Biology, Center for Ocean Mega-Science, Institute of Oceanology, Chinese Academy of Sciences, Qingdao 266071, China; chenghaomiao18@mails.ucas.ac.cn; 2 Laboratory for Marine Biology and Biotechnology, Pilot Qingdao National Laboratory for Marine Science and Technology, Qingdao 266237, China; 3 University of Chinese Academy of Sciences, Beijing 100049, China; 4 Institut de Biologie de l’ENS (IBENS), Département de Biologie, École Normale Supérieure, CNRS, INSERM, Université PSL, 75005 Paris, France; cbowler@biologie.ens.fr; 5 Division of Glycoscience, Department of Chemistry, School of Engineering Sciences in Chemistry, Biotechnology and Health, Royal Institute of Technology (KTH), AlbaNova University Centre, 10691 Stockholm, Sweden; xiaohui.xing@canada.ca (X.X.); bulone@kth.se (V.B.); 6 Australian Research Council Centre of Excellence in Plant Cell Walls, School of Agriculture, Food and Wine, University of Adelaide, Waite Campus, Urrbrae 5064, Australia; 7 Adelaide Glycomics, School of Agriculture Food and Wine, University of Adelaide, Waite Campus, Urrbrae 5064, Australia; 8 State Key Laboratory of Bioactive Seaweed Substances, Qingdao Bright Moon Seaweed Group Co., Ltd., Qingdao 266400, China

**Keywords:** PacBio sequencing, full-length transcriptome, *Thalassiosira weissflogii*, chitin, chitosan

## Abstract

β-Chitin produced by diatoms is expected to have significant economic and ecological value due to its structure, which consists of parallel chains of chitin, its properties and the high abundance of diatoms. Nevertheless, few studies have functionally characterised chitin-related genes in diatoms owing to the lack of omics-based information. In this study, we first compared the chitin content of three representative *Thalassiosira* species. Cell wall glycosidic linkage analysis and chitin/chitosan staining assays showed that *Thalassiosira weissflogii* was an appropriate candidate chitin producer. A full-length (FL) transcriptome of *T. weissflogii* was obtained via PacBio sequencing. In total, the FL transcriptome comprised 23,362 annotated unigenes, 710 long non-coding RNAs (lncRNAs), 363 transcription factors (TFs), 3113 alternative splicing (AS) events and 3295 simple sequence repeats (SSRs). More specifically, 234 genes related to chitin metabolism were identified and the complete biosynthetic pathways of chitin and chitosan were explored. The information presented here will facilitate *T. weissflogii* molecular research and the exploitation of β-chitin-derived high-value enzymes and products.

## 1. Introduction

**Figure 1:**
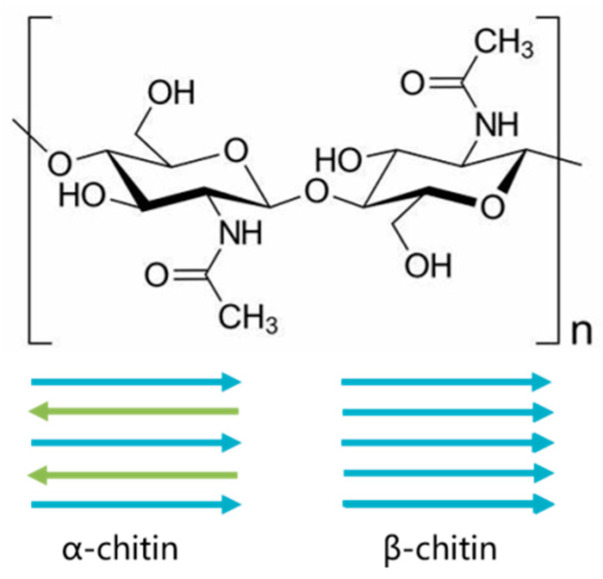
Structure of α-chitin and β-chitin. Anti-parallel arrangement of chitin chains in α-chitin whereas parallel arrangement for β-chitin.

Chitin, a polymer of 1,4-linked β-D*-N*-acetylglucosaminyl residues, is the second most abundant natural biopolymer after cellulose, and is widely distributed across taxa [1,2,3]. Together with its partially de-*N*-acetylated derivative chitosan, chitin has many biomedical applications, such as wound healing, artificial organs and drug delivery [4]. The parallel arrangement of chitin chains in the β-chitin allomorph confers specific properties to the polymer, such as higher solubility, reactivity and swelling compared to the most abundant form of chitin, α-chitin (Figure 1) [5,6]. There are very few classes of organisms that produce β-chitin, such as the diatom *Thalassiosira* sp. [7,8,9]. Various diatoms have been previously reported to produce chitin, mainly in the genera *Thalassiosira* and *Cyclotella* [10,11]. The first report of the occurrence of chitin in diatoms was in *T. weissflogii* (*fluviatilis*), which showed that *T. weissflogii* chitin represented up to 34% of the total cell mass (including the silica) [12]. The content and structure of chitin have been widely studied in diatoms, but its biosynthetic pathway based on the sequencing and gene annotation is incomplete [13,14]. In this study, we first measured the chitin content in the cell walls of three diatom species, *Thalassiosira rotula*, *T. pseudonana* and *T. weissflogii*, and found that *T. weissflogii* had the highest chitin content. However, apart from the identification of chitinous fibres [9], our knowledge of chitin in *T. weissflogii* is limited, which impedes the exploitation of chitin and chitin-related enzymes from this class of organisms.

Diatoms are a major group of phytoplankton and contribute approximately 20% of global primary productivity [15,16]. The last two decades have witnessed a surge in diatom gene information, with complete genomes [17,18,19] and a number of transcriptomes sequenced [20,21,22]. By contrast, *T.* *weissflogii* genes can only be referred to the transcripts released by the Marine Microbial Eukaryote Transcriptome Sequencing Project (MMETSP) [23], and no full-length reference database is openly available so far. Genes encoding enzymes metabolising high-value products such as β-chitin and the full potential of *T. weissflogii* remain to be unveiled. Pacific BioSciences (PacBio) single-molecule real-time (SMRT) sequencing enables the discovery of novel genes and gene product isoforms. It provides longer reads, making them suitable for biological problems that are poorly solved by second-generation sequencing (SGS) [24]. A full-length (FL) transcriptome obtained by PacBio sequencing is an alternative complete transcript assembly for a non-model species. In the present study, the FL transcriptome of *T. weissflogii* was sequenced to elucidate the genetic profile and uncover the chitin-related genes of this diatom species. Our study highlights information for β-chitin metabolism in *T. weissflogii,* provides a reference genetic dataset for future studies and assists in evolutionary studies to a broad taxonomy. The FL transcriptome will enable the construction of chitin metabolic pathways and facilitate the in vitro application of chitin-related enzymes.

## 2. Results and Discussion

### 2.1. Glycosidic Linkage Analysis

Three representative species of the *Thalassiosira* genus were selected and subjected to glycosidic linkage analysis of cell wall polysaccharides (Table 1). Glucosyl (Glc) and *N*-acetylglucosaminyl (GlcNAc) residues were the two most abundant monosaccharides in all three *Thalassiosira* species analysed. Notably, *T. weissflogii* contained more GlcNAc than the other two species, representing 11.5 and 4.9 times that in *T. pseudonana* and *T. rotula*, respectively (Table 1). This indicates that *T. weissflogii* might be the most appropriate candidate of all three species analysed for exploring chitin-related enzymes in diatoms.

### 2.2. Staining of Chitin and Chitosan

**Figure 2:**
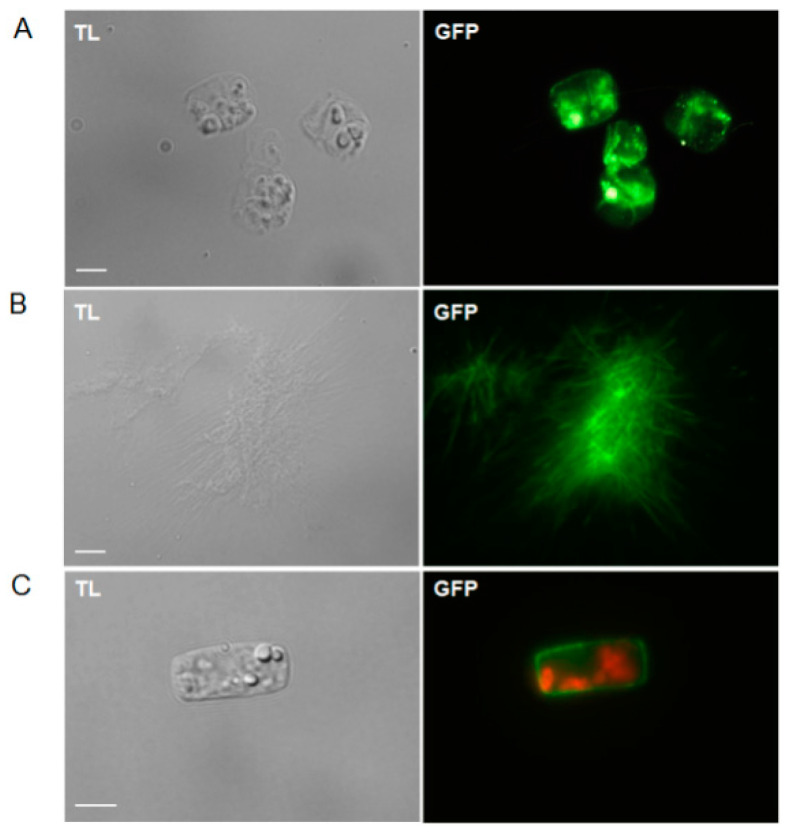
Fluorescence observations indicating the presence of chitin and chitosan in *T. weissflogii*; (**A**) Cells stained with CBP-eGFP; (**B**) Chitin microfibrils observation after CBP-eGFP staining; (**C**) Cells stained with CAP-eGFP. TL: transmission light; GFP: green fluorescent protein. Scale bar = 5 μm.

In order to observe the localisation of chitin and chitosan, chitin-binding protein (CBP) and chitosan-affinity protein (CAP) tagged with enhanced green fluorescence protein (eGFP) were used to stain *T. weissflogii* live cells. The results showed that the fluorescence of chitin was present in the cytoplasm and at the cell boundary (Figure 2A). Notably, strong, clumped fluorescent signals were detected in the cell suspension (Figure 2B), which indicated that a large quantity of chitin detached from the cell and aggregated into clusters. This is the evidence that *T. weissflogii* could produce a large amount of external chitin microfibrils, which was consistent with the scanning electron microscopy-derived results of Ogawa et al. (2011) [9]. After CAP-eGFP staining, continuous fluorescence appeared around the cells (Figure 2C), which is a new discovery indicating that *Thalassiosira* can synthesise chitosan in the cell wall.

### 2.3. Sequencing and Data Processing

**Figure 3:**
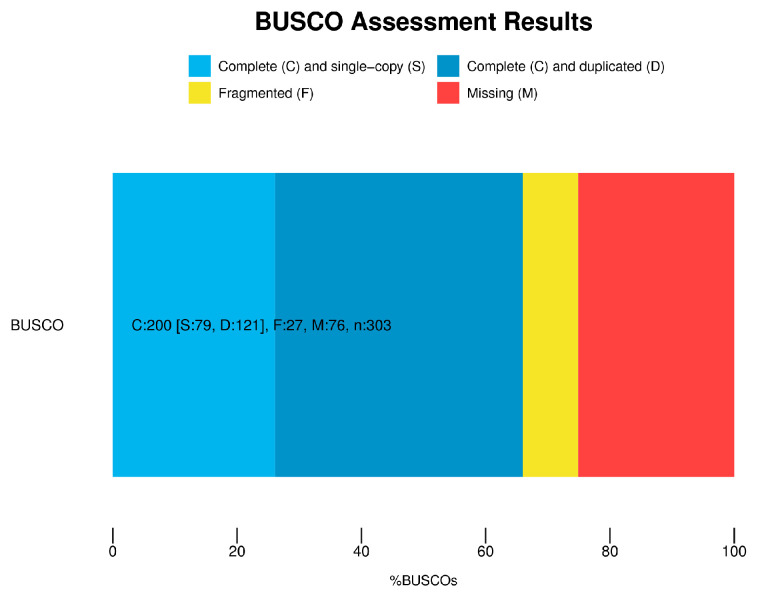
Results of BUSCO analysis. “C”: Complete; “S”: Single-copy; “D”: Duplicated; “F”: Fragmented; “M”: Missing.

As a source of high chitin content, *T. weissflogii* was subjected to PacBio sequencing. A total of 70,038,125,361 basepairs (bp) containing 44,233,932 subreads were yielded (Table 2). In total, 1,021,310 circular consensus sequences (CCSs) with an average length of 2127 bp were generated after merging transcripts with at least two full passes. The full-length non-chimeric (FLNC) sequences of the CCSs were then clustered and polished, producing 110,527 high-quality isoforms and 338 low-quality isoforms. After removing the sequence redundancy, the polished high-quality isoforms were trimmed to 25,412 unigenes for further analyses (Table 2). Only 25,412 (2.5%) of the 1,021,310 CCSs were retained as unigenes after redundancy removal, which was less than the transcript numbers of the two *T. weissflogii* strains released in the MMETSP project (55,443 of strain CCMP1336 and 282,372 of strain CCMP1010) [23]. The significant level of redundancy showed the good depth and integrity of the *T. weissflogii* FL transcriptome achieved via PacBio sequencing technology. The GC content of the *T. weissflogii* FL transcriptome was comparable to those of *T. rotula* and three *Pseudo-nitzschia* species [25,26], indicating high homogeneity within diatoms. A relatively high percentage of complete Benchmarking Universal Single-Copy Orthologs (BUSCOs), 200 out of 303 total BUSCOs (66.01%), showed the high-quality assembly completeness of our transcriptomes (Figure 3).

Transcriptomes reflect gene expression potentially affecting the physiological and biochemical processes from a molecular perspective. In diatoms, SGS has revealed genetic information in terms of underwater adhesion, biofuel accumulation and nitric oxide synthase genes in *Amphora coffeaeformis, Fistulifera solaris* and *Pseudo-nitzschia* [26,27,28]. The PacBio sequencing captures full-length transcripts without assembly and overcomes the limitations of SGS, such as complex genomic region assembly and determination, isoform and methylation detection [24]. For a non-model diatom without a reference genome, SGS sequencing is inadequate for producing an FL transcriptome. PacBio sequencing has been used to analyse the FL transcriptome of animals and higher plants [29,30]. The *T. weissflogii* FL transcriptome here represents the first general transcription encyclopaedia of the species and FL transcriptome from a diatom. The average read length of the *T. weissflogii* FL transcriptome obtained by PacBio sequencing was longer than those of other diatom species acquired by SGS [20,27]. Furthermore, compared with the SGS transcriptome of *T. rotula*, the *T. weissflogii* FL transcriptome contained higher N50 and longer transcripts, and retained much fewer genes [25].

### 2.4. Analyses of Coding Sequence, Long Non-Coding RNAs and Transcription Factors

**Figure 4:**
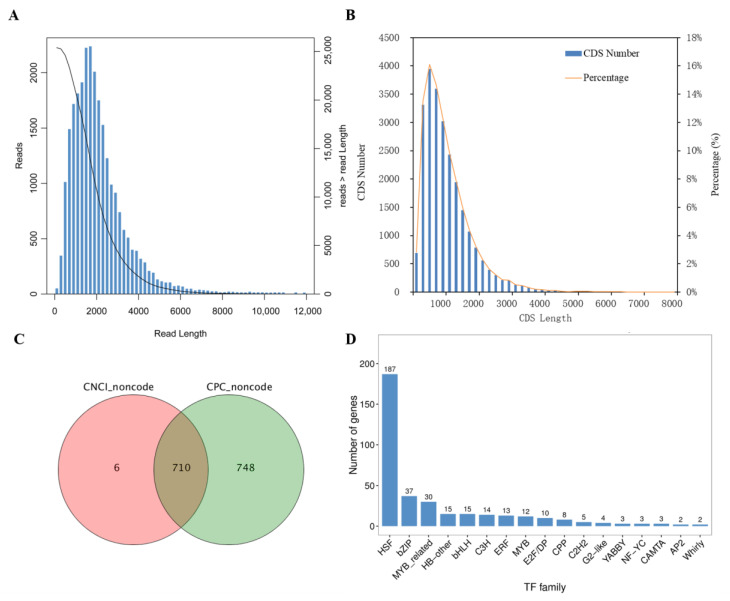
Composition of the *T. weissflogii* FL transcriptome; (**A**) Length distribution of unigenes; (**B**) Length distribution of CDSs; (**C**) LncRNA numbers predicted by CNCI and CPC; (**D**) Numbers of top ten TF families.

The coding sequence (CDS) of a gene is a singular section of DNA or RNA that encodes the corresponding protein. In 25,412 unigenes with an average length of 2045.04 bp (Figure 4A), a total of 24,500 (96.4%) CDSs were predicted, with the most represented length range being 401–600 bp (Figure 4B). LncRNAs are defined as transcripts longer than 200 nucleotides that are not translated into protein. They are fundamentally involved in biological processes such as transcription, translation, protein localisation, cellular structure integrity, reprogramming and other cellular activities [31]. Altogether, 710 unigenes were characterised as lncRNAs by the joint prediction of CNCI and CPC (Figure 4C). TFs are proteins that recognise and bind specific nucleotide sequences, mediating the primary expression of nearby genes during transcription [32]. A total number of 363 TF unigenes were identified and categorised into 17 families, the top ten of which are shown in Figure 4D. Over half of the TFs were members of the heat shock transcription factor (HSF) family (187, 51.5%). The proportion of HSF in the TFs of *T. weissflogii* was even higher than that reported in the *Phaeodactylum tricornutum* and *T. pseudonana* TFs, where the HSF family constituted the most abundant TF class (33.0% and 36.4%, respectively) [33]. The number of TF genes identified in this FL transcriptome was higher than those reported in the *P. tricornutum* and *Nitzschia* SGS transcriptomes, although the TFs of *Nitzschia* were sorted into 38 families [20,34].

### 2.5. Annotation Analyses

**Figure 5:**
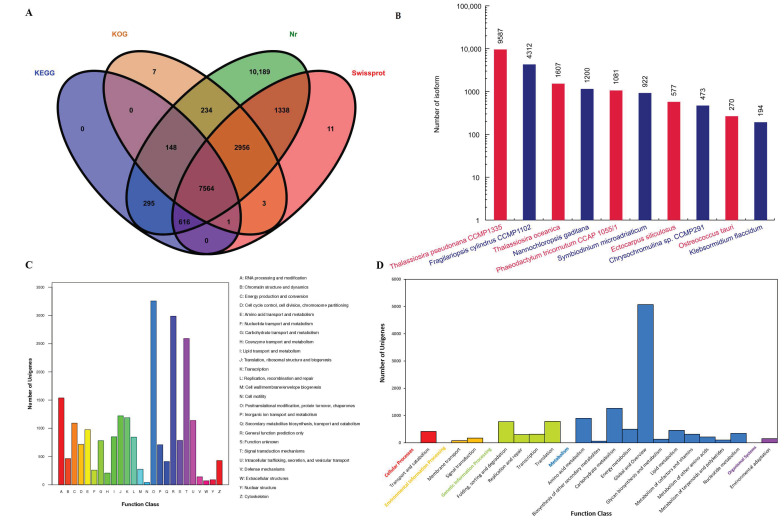
Annotation information of *T. weissflogii* FL transcriptome unigenes; (**A**) Annotation summary from the NR, Swiss-Prot, KEGG and KOG databases; (**B**) Species distribution annotation from the NR database; (**C**) Functional annotation from the KOG database; (**D**) Functional annotation from the KEGG database.

A total of 23,362 unigenes were annotated (91.9%), of which 7564 could be annotated in all four of the Non-Redundant Protein Sequence Database (NR), Swiss-Prot, euKaryotic Ortholog Groups (KOG) and Kyoto Encyclopedia of Genes and Genomes (KEGG) databases. The number of unigenes assigned annotation terms in the above four databases were 23,340, 12,489, 10,913 and 8624, respectively (Figure 5A). According to the prediction by the NR database, 23,340 unigenes were annotated in 189 homologous species, where the top ten species are shown in Figure 5B. *T. pseudonana*, whose genome was the first to be described for a eukaryotic marine phytoplankton [17], was found to contain the highest number of homologous sequences (9587, 41.1%) with *T. weissflogii*. The second was a pennate diatom *Fragilariopsis cylindrus* and the third was the centric diatom *Thalassiosira oceanica.* This suggested that *T. weissflogii* might be phylogenetically more closely related to *F. cylindrus* than its centric companion. Two macroalgal species, *Ectocarpus siliculosus* and *Klebsormidium flaccidum*, were ranked among the top ten species.

From the KOG analysis, 10,913 unigenes were categorised into 25 classes; the most prominent class was “posttranslational modification, protein turnover, chaperones” (Figure 5C). A total of 8624 unigenes were annotated in 127 pathways in the KEGG database, with five A classes and nineteen B classes (Figure 5D). The B classes “carbohydrate metabolism” and “amino acid metabolism” were at the second and third places, which demonstrated the pivotal roles of these relevant genes for *T. weissflogii*. Within marine phytoplankton, diatoms outcompeted others for ample NO_3_, which fuels CO_2_ fixation and the proliferation of diatoms in nutrient-rich environments such as upwellings [35,36]. These relevant genes might enable diatoms to rapidly respond to variations in carbon and nutrient sources and contribute to their ecological success.

### 2.6. Analyses of Alternative Splicing and Simple Sequence Repeats

**Figure 6:**
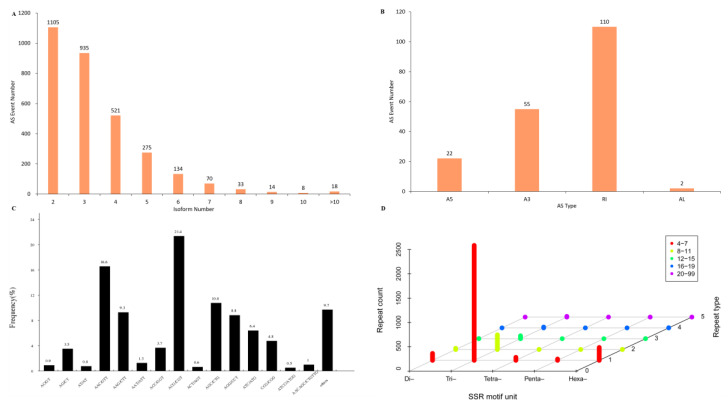
AS events and SSRs of *T. weissflogii* FL transcriptome; (**A**) Alternatively spliced isoform distribution; (**B**) AS types identified in the FL transcriptome, including A3 (alternative 3′ splice sites), A5 (alternative 5′ splice sites), AL (alternative last exons) and RI (retained intron); (**C**) Percentages of SSR motifs; (**D**) 3D histogram of SSR components. Di- stands for dinucleotide repeats, Tri- for trinucleotide repeats, Tetra- for tetranucleotide repeats, Penta- for pentanucleotide repeats, Hexa- for hexanucleotide repeats.

The AS process provides eukaryotes with peculiarly versatile means of genetic regulation. Splicing site alterations from a gene generates multiple mRNA and protein products [37]. In the present study, 3113 AS events were detected, with the genes containing two isoforms ranked the highest (1105) (Figure 6A). Only 189 events were classified into four AS types (Figure 6B), and the major AS type was the retained intron, which was previously reported in *P. tricornutum* [38]. No exonskipping, mutually exclusive exons and alternative first exon AS events were detected. However, the detection of AS events in this study was limited due to the lack of a reference genome of *T. weissflogii*. This would result in the missing and underestimation of some types of AS [29]. An SSR or microsatellite is a repetitive DNA sequence where certain motifs are repeated. A total of 3295 SSRs were detected (Figure 6C), exceeding the SSRs identified in *P. tricornutum* on numbers (1135 in Phatr2 and 255 in Phatr3) [38]. In these SSRs, the largest group was trinucleotide repeats (2754, 83.6%), most of which were 4–7 repeats (Figure 6D). SSRs are highly polymorphic genetic markers [39], and the information here will be of convenience for phylogenetic studies of diatoms.

### 2.7. Chitin-Related Gene Mining

Transcriptome profiling has revealed information of chitin-related genes in many organisms, e.g., a chitin elicitor receptor kinase gene in barley [40], chitin utilisation-related genes in *Vibrio coralliilyticus* and *Photobacterium galatheae* [41] and chitin metabolism-related genes in *Glyphodes pyloalis* Walker [42]. To date, reports identifying chitin-related genes on whole-genome or -transcriptome scales in diatoms are scarce. Traller et al. (2016) compiled chitin metabolism pathway genes in *Cyclotella cryptica* [14]. Cheng et al. (2021) characterised a gene family of 24 members encoding chitinases in *T. pseudonana* [43]. In contrast, the identification of these classes of genes in *T. weissflogii* at the whole-genome or -transcriptome level has been lacking.

**Figure 7:**
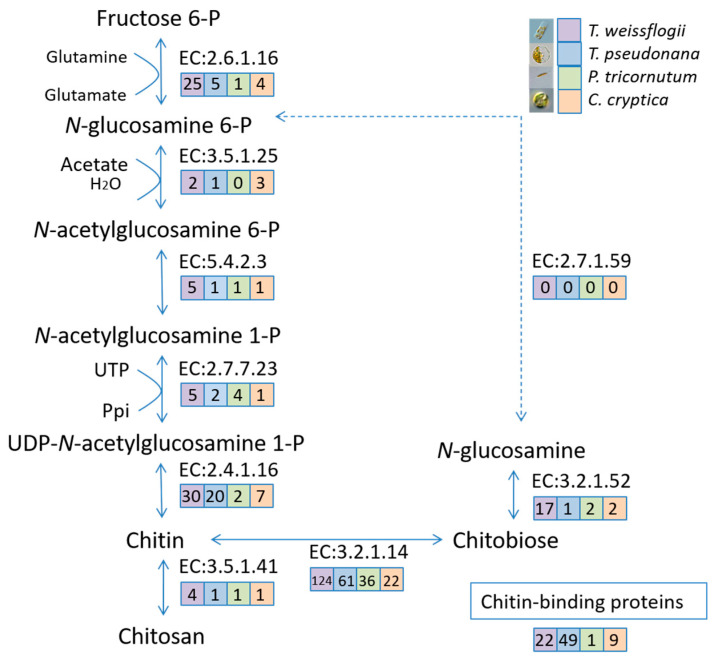
Hypothetical chitin metabolism pathways in four diatom species. Enzymes are represented by EC numbers: Glutamine--fructose-6-phosphate transaminase (isomerising) (EC:2.6.1.16), *N*-acetylglucosamine-6-phosphate deacetylase (EC:3.5.1.25), Phosphoacetylglucosamine mutase (EC:5.4.2.3), UDP-*N*-acetylglucosamine diphosphorylase (EC:2.7.7.23), Chitin synthase (EC:2.4.1.16), Chitinase (EC:3.2.1.14), Chitin deacetylase (EC:3.5.1.41), Beta-*N*-acetylhexosaminidase (EC:3.2.1.52), *N*-acetylglucosamine kinase (EC:2.7.1.59). Numbers of identified enzyme-encoding genes are represented in differently coloured boxes. Enzyme-encoding genes unidentified in the *T. weissflogi* FL transcriptome are indicated by dashed lines. The schematic was adapted from Traller et al. (2016) [14].

From the FL transcriptome dataset of *T. weissflogii*, 234 unigenes potentially related to chitin metabolism were identified, including 25 glutamine-fructose-6-phosphate transaminases (isomerising), 2 *N*-acetylglucosamine-6-phosphate deacetylases, 5 phosphoacetylglucosamine mutases, 5 UDP-*N*-acetylglucosamine diphosphorylases, 30 chitin synthases, 124 chitinases, 17 beta-*N*-acetylhexosaminidases, 4 chitin deacetylases and 22 chitin-binding proteins (Figure 7). We constructed the chitin metabolism pathway in *T. weissflogii* and compared it with pathways in *P. tricornutum*, *T. pseudonana* and *C. cryptica*. We found that all four diatom species harbour rather complete chitin metabolism pathways (Figure 7). The total number of identified chitin-related genes in *T. weissflogii, T. pseudonana, C. cryptica* and *P. tricornutum* were 234, 141, 50 and 48, respectively. The higher abundance of these genes in *T. weissflogii*, particularly the chitin synthase, chitinase and chitin deacetylase genes that are directly related to chitin, implies a more active chitin metabolism in *T. weissflogii.* As *P. tricornutum* does not generate chitin fibrils, it is not surprising that it harbours the lowest number and fewest types of chitin-related genes. The *N*-acetylglucosamine kinase that phosphorylates GlcNAc to GlcNAc-6-P was absent in all four species. We speculate that this may be because diatoms lack the GlcNAc phosphorylation process, rather than due to inconclusive annotations or distinction from characterised *N*-acetylglucosamine kinases, as Traller et al. (2016) suggested [14]. In *T. weissflogii*, chitinases accounted for over half of the sum (53.0%), indicating the presence of an active chitin degradation process. However, these genes identified in the FL transcriptome require further sequence analyses for each isoform, which is needed for a better and more accurate understanding of the entire chitin metabolic pathway.

## 3. Materials and Methods

### 3.1. Glycosidic Linkage Analysis

*T. weissflogii*, *T. pseudonana* and *T. rotula* cultures were grown at 19 °C with 12 h:12 h light:dark cycles (100 μmol m^−2^ s^−1^). Approximately 100 mg lyophilised samples were collected from large-scale cultures of each *Thalassiosira* species for glycosidic linkage composition analysis. The cell wall sample preparation and linkage analysis (methylation–GC–MS analysis) was performed as described by Shao et al. (2019) [6]. Experiments were conducted in duplicate.

### 3.2. Staining of Chitin and Chitosan

*T. weissflogii* cell cultures at exponential period were centrifuged at 13,000× *g*, prior to a suspension of cell pellets in fresh f/2 liquid medium. CBP-eGFP and CAP-eGFP were used to visualise the presence and localisation of chitin and chitosan, respectively, according to the methods by Hardt and Laine (2004) and Nampally et al. (2012) [44,45]. GFP signal was observed using a Zeiss Axio Imager M2 fluorescent microscope (Zeiss, Germany). ImageJ software was used to analyse the epifluoresent photos.

### 3.3. T. weissflogii Collection and RNA Extraction

*T. weissflogii* (9021) for sequencing was acquired from the Microalgae Collection Center at Ningbo University, Ningbo, China, and grown in optimised f/2 liquid medium provided by Shanghai Guangyu Biological Technology Co., Ltd., Shanghai, China. Cells were cultured at 19 °C with 12 h:12 h light:dark cycles (100 μmol m^−2^ s^−1^) and swirled at 100 rpm. Cells reaching the exponential phase were harvested and frozen in liquid nitrogen and stored at −80 °C for further experiments. Total RNA was extracted by grinding the *T. weissflogii* frozen sample in TRIzol reagent (Life Technologies, Carlsbad, CA, USA) and processed following the protocol provided by the manufacturer.

### 3.4. Library Construction and Sequencing

RNA concentration was checked using a Nanodrop micro-spectrophotometer (Thermo Fisher Scientific, Waltham, MA, USA) and RNA integrity was verified with a Bioanalyzer 2100 (Agilent Technologies, Palo Alto, CA, USA). mRNA was enriched using Oligo (dT) magnetic beads and then reverse-transcribed into cDNA using the Clontech SMARTer PCR cDNA Synthesis Kit (Clontech, Palo Alto, CA, USA). The cDNA was then amplified by PCR. All cDNAs were DNA damage-repaired, end-repaired and connected with adaptors. PacBio sequencing was then conducted on a PacBio Sequel platform (Gene Denovo Biotechnology Co., Guangzhou, China).

### 3.5. Data Processing

The raw data were initially processed following the SMRT Link v6.0 standard pipeline [46]. The offline transcripts with full passes ≥2 were merged into CCSs. FLNC sequences of CCSs were then clustered and corrected by the interactive clustering and error correction (ICE) algorithm. The corrected isoforms were aligned with non-full-length transcripts using Quiver algorithm, generating polished consensus isoforms of high- and low-quality. The software CD-HIT-v4.6.7 was used to remove the redundancy of the polished high-quality isoforms by merging the sequences with a threshold of 99% identities, ultimately obtaining the FL transcriptome (unigenes). The assembly completeness was assessed by BUSCO software using Eukaryota dataset of OrthoDB [47].

### 3.6. Functional Annotation

All of the unigenes obtained were functionally annotated by BLASTx analysing against the NR, Swiss-Prot, KEGG and KOG databases with an E-value < 1 × 10^−5^. Each unigene was annotated with the information of the protein with the highest sequence similarity. GO annotation was performed using the Blast2GO software with the NR annotation results [48]. Unigenes with the first 20 highest scores and ≥33 high-scoring segment pair (HSPs) hits were selected to undergo Blast2GO analysis. Subsequently, the WEGO software was used to classify the functional annotation of the unigenes [49].

### 3.7. Gene Type Analyses

The unigenes were blastx searched against the databases with the E-values < 1 × 10^−5^ to retrieve a protein sequence for each unigene from either of the four databases in the order of NR, Swiss-Prot, KEGG and KOG, which then located the CDS of the unigene. The unigenes that failed to retrieve a protein sequence were subjected to ANGEL for CDS prediction [50]. LncRNA analysis was conducted on the unigenes that were not annotated to the four databases by combining the prediction of Coding-Non-Coding Index (CNCI) and Coding Potential Calculator (CPC) software [51,52]. Unigenes predicted as non-coding by both CNCI and CPC were considered lncRNAs. TF analysis was performed using hmmscan against the Plant TFdb database [53].

### 3.8. Detection of Alternative Splicing and Simple Sequence Repeats

The AS events were identified using the software Cogent and SUPPA [54,55]. Cogent partitioned high-quality FLNC sequences into gene families by K-mer = 30 and K-mer similarity >95%, and built each family a reference sequence by De Bruijn graph methods. Then, the AS events were detected using SUPPA with references. The software MISA was employed to identify SSRs within the FL transcriptome with the parameters of length-minimum number of repetitions = 2–6 or 3–5 or 4–4 or 5–4 or 6–4 and interruptions of 100 bp [56].

### 3.9. Mining of Chitin Metabolism Genes in Diatoms

Chitin-related genes in the *T. weissflogii* FL transcriptome were annotated by the four databases of NR, Swiss-Prot, KEGG and KOG. These genes in the genomes of *P. tricornutum* and *T. pseudonana* were retrieved from the Joint Genomics Institute PhycoCosm database (JGI PhycoCosm, https://jgi.doe.gov/data-and-tools/phycocosm/, accessed on 20 April 2021). Gene retrieval was performed by searching with enzyme names on the ENZYME database (https://enzyme.expasy.org/, accessed on 20 April 2021) and with genes published in previous literature as a supplement. Numbers of the corresponding genes in *C. cryptica* were obtained from Traller et al. (2016) [14].

## 4. Conclusions

In this study, we constructed a full-length transcriptome of *T. weissflogii* using PacBio sequencing. The transcriptome consists of 25,412 unigenes, 23,362 annotated unigenes, 710 lncRNAs, 363 TFs, 3113 AS events and 3295 SSRs. Furthermore, we identified 234 genes related to chitin metabolism. The whole metabolic pathway of chitin biosynthesis and degradation was explored. The information published here will pave the way for *T. weissflogii* molecular research in the future, expand the resource of β-chitin and promote the development of high-value enzymes.

## Figures and Tables

**Table 1:** Glycosidic linkage composition of the three *Thalassiosira* microalgae samples.

No.	Linkage	Composition (Mol%)		
		Tw	Tp	Tr
1	4-Glc*p*	15.5	25.4	4.3
2	3-Glc*p*	12.8	27.8	56.4
3	4-GlcNAc*p*	6.9	0.6	1.4
4	t-Gal*p*	6.7	2.2	3.1
5	2,3-Glc*p*	5.5	1.5	2.0
6	t-Man*p*	5.4	1.7	1.7
7	2-Man*p*	4.9	2.4	3.1
8	4-Xyl*p*	3.7	1.9	1.4
9	t-Xyl*p*	3.3	2.9	0.8
10	6-Man*p*	2.9	ND	ND

Tw: *Thalassiosira weissflogii*; Tp: *Thalassiosira pseudonana*; Tr: *Thalassiosira rotula*; ND means “not detected”.

**Table 2:** Summary of the *T. weissflogii* FL transcriptome statistics.

Statistical Data	*T. Weissflogii*	
Raw reads	Subread number	44,233,932
	Average length (bp)	1583
	N50 (bp)	2003
CCSs	Number of reads Number of CCS bases CCS read length (mean) (bp) Number of passes (mean)	1,021,310 2,172,901,990 2127 8
Clustered reads	Number of polished high-quality isoforms	110,527
	Number of polished low-quality isoforms	338
Unigenes	Total number	25,412
	Total length (bp)	51,968,546
	Maximum length (bp)	11,939
	Minimum length (bp)	64
	Average length (bp)	2045.04
	N50 length (bp)	2417
	GC content	46.95%

## Data Availability

The obtained raw data of the *T. weissflogii* FL transcriptome were deposited into the NCBI SRA database with the accession number PRJNA717330. Transcripts of *T. weissflogii* in MMETSP are openly available on the iMicrobe website with the accession number CAM_P_0001000 (https://www.imicrobe.us/, accessed on 15 February 2021).
